# Intraoperative traction has a negligible time-dependent influence on patient-reported outcomes after hip arthroscopy: a cohort study

**DOI:** 10.1093/jhps/hnad034

**Published:** 2023-11-15

**Authors:** Jacob D Feingold, Thacher Ryan R., Adit Maniar, Stefan Mitrasinovic, Samarth Venkata Menta, Anil Ranawat

**Affiliations:** Sports Medicine Institute, The Hospital for Special Surgery, 535 E 70th St, New York, NY 10021, USA; Sports Medicine Institute, The Hospital for Special Surgery, 535 E 70th St, New York, NY 10021, USA; Department of Orthopaedics, London Health Sciences Centre, 339 Windermere, London, ON N6B, Canada; Sports Medicine Institute, The Hospital for Special Surgery, 535 E 70th St, New York, NY 10021, USA; Sports Medicine Institute, The Hospital for Special Surgery, 535 E 70th St, New York, NY 10021, USA; Sports Medicine Institute, The Hospital for Special Surgery, 535 E 70th St, New York, NY 10021, USA

## Abstract

The aim of this study is to determine if post-operative patient-reported outcome measures (PROMs) are influenced by hip arthroscopy traction duration.

Patients from a local prospective hip arthroscopy database were retrospectively analyzed. Four hip-specific PROMs were utilized: modified Harris Hip Score (mHHS), Hip Outcome Score—Activities of Daily Living (HOS-ADL), Hip Outcome Score—Sports Specific (HOS-SS), and international Hip Outcome Tool (iHOT). PROMs were collected pre-operatively and 6 months, 1 year and 2 years post-operatively. Two cohorts were created based on a cut-off corresponding to the 66th percentile for our patient cohort. Analyses were completed for each PROM at each post-operative interval with univariable statistics. Multivariable statistics were examined to identify the variables that were predictive of achieving post-operative minimal clinically important difference (MCID) at the 2-year follow-up.

Overall, 222 patients met the inclusion criteria. The mean age was 32.4 ± 9.4 years, and 116 (52.3%) were female. The average traction time of the study population was 46.1 ± 12.9 min. A total of 145 patients were included in the short traction cohort (65%) with traction times of <50 min (66th percentile). No significant differences were found regarding PROM scores or MCID achievement rates between both cohorts at any post-operative period. In multivariable analyses, achievement of MCID was predicted by a decrease in traction time for all PROMs and pincer-type resection for mHSS, HOS-ADL and iHOT.

There was no difference in PROMs and MCID achievement between longer and shorter traction time cohorts. On multivariable analysis, a decrease in traction time is predictive of MCID for all PROM scores and pincer-type resection was predictive of MCID for most PROM scores.

**Level of evidence:** Level III, cohort study

## INTRODUCTION

Hip arthroscopy is a well-accepted alternative to surgical hip dislocation for the treatment of a variety of intra-articular hip disorders. The use of gross traction during hip arthroscopy is necessary to allow for the introduction of the arthroscope and instruments but has been associated with a wide range of complications, including sciatic or femoral nerve neuropraxias, perineal contusions, knee pain and traction boot injuries [1–5]. The majority of these problems resolve after a few weeks; however, in extreme cases, it can cause semi-permanent to permanent disabilities [3]. Furthermore, studies have demonstrated an association between gross traction and rising levels of creatine kinase and d-dimer [6, 7]. These studies suggest physiologic changes that may expose patients to systemic complications, although no clinical correlation is yet made. Decreasing the overall weight used for traction and reducing the total traction times have been described as methods to limit these complications [1, 5].

Despite the growing body of literature associating traction with post-operative complications in hip arthroscopy, to our knowledge, no studies to date have investigated the relationship between overall traction time and patient-reported outcome measures (PROMs). Earlier studies have validated the use of a number of PROMs and defined a minimal clinically important difference (MCID) for patients undergoing hip arthroscopy for femoroacetabular impingement (FAI) [8–10].

To expand upon the growing body of literature surrounding hip arthroscopy outcomes, the purpose of this study is to determine if post-operative PROMs are influenced by hip arthroscopy traction duration. The primary hypothesis predicted inferior post-operative outcome measures in the cohort composed of patients with extended the traction duration. Secondary hypotheses included the following: (i) cohorts with the longer traction duration would meet the post-operative MCID at comparatively lower rates and (ii) incremental increases in traction time would independently increase the risk of failing to achieve the MCID.

## MATERIALS AND METHODS

This study received institutional review board (IRB) approval from our institution. All patient data utilized in the present study were collected prospectively from 2006 to 2018 via a separate IRB-approved multi-surgeon hip registry. The registry is responsible for collecting patient demographic information and treatment-related data. It also tracks clinical outcomes for patients undergoing hip arthroscopy for FAI. The current study included patients from six high-volume fellowship-trained hip preservation surgeons.

PROMs were collected pre-operatively and at set time intervals after hip arthroscopy. Four hip-specific PROMs were utilized: (i) the modified Harris Hip Score (mHHS) [11], (ii) Hip Outcome Score—Activities of Daily Living (HOS-ADL) [10], (iii) Hip Outcome Score—Sports Specific (HOS-SS) [8] and (iv) the international Hip Outcome Tool (iHOT) [12]. PROMs were collected after surgery at three time points: (i) 6 months, (ii) 1 year and (iii) 2 years post-operatively. Analyses concerning both absolute PROM scores and comparisons to established MCID values enabled an assessment of arthroscopic efficacy. Threshold values for MCID achievement published in prior literature were applicable to the present study and used for comparative purposes [9].

Patients who underwent hip arthroscopy for FAI were considered valid for inclusion if they had both a pre- and post-operative score available for at least one of the PROMs. Patients were excluded from the analysis if any of the following criteria were met: (i) history of ipsilateral hip surgery, (ii) ipsilateral hip osteoarthritis (defined as Tonnis grade >1), (iii) simultaneous bilateral hip arthroscopy and (iv) hip arthroscopy in conjunction with another ipsilateral hip surgery (i.e. hip arthroscopy with concurrent periacetabular osteotomy).

After the application of the abovementioned criteria, 222 patients were included in the study. Pre-operative scores were available for all patients. Follow-up compliance rates on PROMs were 100% for 6 months and 1 year post-operatively and 80% for 2 years post-operatively, which are similar to compliance rates reported on PROMs in national arthroscopic registries [13].

Traction times were derived through the assessment of operative records via a chart review. The distribution of traction time across the study population provided the basis for two different study cohorts. The average traction time of the study population was 46.1 ± 12.9 min. Overall, 145 patients (65%) with a traction time below the 66th percentile of the study population (<50 min) were designated to the short traction cohort. Seventy-seven patients (35%) were included in the long traction cohort (≥50 min).

### Surgical technique

The senior author’s preferred surgical technique is described here. This study included patients from six high-volume fellowship-trained hip preservation surgeons and may have had variations in surgical technique. After applying traction, a lateral para-trochanteric and mid-anterior portal is created with the help of fluoroscopy. Next, a diagnostic arthroscopy is performed. An interportal cut is created for better visualization. A separate anterolateral accessory portal is created as required. After preparation of the labrum and bone, suture anchors are used with a simple suture passed through the labrum to recreate normal labral anatomy. Next, traction is released and the arthroscope is placed in the peripheral compartment. A T capsulotomy is made to better visualize the cam lesion. Next, a cam decompression is performed using a shaver and burr and confirmed on fluoroscopy. The capsule and interportal cut are then closed using non-absorbable sutures.

### Statistical analysis

Descriptive statistics were reported as means and standard deviations and frequencies and percentages where appropriate. For multivariable analyses, logistic regression models were fit for categorical variables: (i) patient sex, (ii) diagnosis of a pincer lesion, (iii) undergoing pincer resection and continuous variables: (iv) traction time and (v) surgical time. A patient was defined to have achieved his post-operative MCID if there was a difference of 8.2 (mHHS), 8.3 (HOS-ADL), 14.5 (HOS-SS) and 12.1 (iHOT). For the models, covariates included preoperative factors reaching *P* < 0.20 on univariate comparisons. These analyses were conducted using Python 3.9.13 and packages pandas 1.5.3, scipy 1.10.0 and statsmodels 0.13.5. *P*-values were two tailed, and an α = 0.05 was considered statistically significant.

## RESULTS

Overall, 3528 patients undergoing hip arthroscopy were included in the prospective registry. Of these, 222 met the inclusion criteria. Baseline characteristics are provided in Table I. The mean age was 32.4 ± 9.4 years. A total of 116 (52.3%) were female. All patients had a diagnosis of a labral tear and cam-type FAI. There was a significant difference in the number of patients with a diagnosis of a pincer-type FAI between the two groups, 17.9% in the short traction and 44.2% in the long traction groups (*P* < 0.05). Patients had a range of procedures done during the surgery, which included labral repair, labral debridement, cam decompression and pincer resection. The only significant difference between the groups was pincer resection, 17.2% in the short traction and 49.4% in the long traction groups (*P* < 0.05).

**Table I.** Univariate analysis comparing pre-operative characteristics and patient demographics between two cohorts based on intraoperative traction time

	*All (n = 222)*	*Short traction (n = 145)*	*Long traction (n = 77)*	*P-value*
Age (years), mean ± SD	32.4 ± 9.4	32.3 ± 8.7	32.7 ± 10.7	0.69
Female, *n* (%)	116 (52.3)	83 (57.2)	33 (42.9)	0.06
Comorbidities, *n* (%)				
Labral tear	222 (100)	145 (100)	77 (100)	1
Cam lesion	222 (100)	145 (100)	77 (100)	1
Pincer lesion	60 (27.0)	26 (17.9)	34 (44.2)	<0.05**
Surgical intervention, *n* (%)				
Labral repair	185 (83.3)	118 (81.4)	67 (87.0)	0.38
Labral debridement	37 (16.7)	27 (18.6)	10 (13.0)	0.38
Cam decompression	222 (100)	145 (100)	77 (100)	1
Pincer resection	63 (28.4)	25 (17.2)	38 (49.4)	<0.05**
Traction time, mean ± SD	46.1 ± 12.9	38.3 ± 6.2	60.7 ± 8.9	<0.05**
Surgical time, mean ± SD	89.9 ± 22.1	78.6 ± 10.5	112.0 ± 22.4	<0.05**

Values do not add up to 100% where there are missing data. The left column represents all eligible patients for the study. The second column represents patients with an intraoperative traction time of <50 min. The third column represents patients with an intraoperative traction time of ≥50 min.

** A significant difference with *P*-values of <0.05.

The average traction time for all included patients was 46.1 ± 12.9 min, while the short and long traction group had 38.3 ± 6.2 and 60.7 ± 8.9 min (*P* < 0.05), respectively. The average surgical time for all included patients was 89.9 ± 22.1 min, while the short and long traction group had 78.6 ± 10.5 and 112.0 ± 22.4 min (*P* < 0.05), respectively.

The average pre- and post-operative PROM scores reported by each cohort are provided in Table II. The rate at which each cohort achieved the post-operative MCID for each PROM is presented in Table III.

**Table II.** Scores on PROMs between two cohorts based on intraoperative traction time

	*All (n = 222)*	*Short traction (n = 145)*	*Long traction (n = 77)*	*P-value*
Baseline, mean ± SD				
mHSS	61.7 ± 12.6	61.2 ± 12.8	62.6 ± 12.2	0.35
HOS-ADL	73.3 ± 16.2	72.7 ± 16.9	74.5 ± 14.9	0.63
HOS-SS	50.8 ± 23.8	50.2 ± 24.1	51.9 ± 23.4	0.55
iHOT	39.1 ± 17.2	38.5 ± 17.5	40.1 ± 16.8	0.62
6 months post-operatively, mean ± SD				
mHSS	82.3 ± 13.1	81.7 ± 14.0	83.3 ± 11.0	0.93
HOS-ADL	89.9 ± 11.2	90.1 ± 11.8	89.6 ± 10.0	0.32
HOS-SS	72.1 ± 24.8	72.7 ± 25.1	71.0 ± 24.4	0.44
iHOT	72.4 ± 19.9	72.1 ± 20.9	72.9 ± 18.2	0.89
1 year post-operatively, mean ± SD				
mHSS	85.2 ± 14.5	86.1 ± 14.0	83.4 ± 15.2	0.22
HOS-ADL	91.7 ± 12.8	92.0 ± 12.7	91.1 ± 13.2	0.38
HOS-SS	81.1 ± 22.8	81.8 ± 23.3	79.7 ± 21.8	0.24
iHOT	77.2 ± 21.1	77.7 ± 21.5	76.3 ± 20.6	0.37
2 years post-operatively, mean ± SD				
mHSS	85.7 ± 14.9	86.0 ± 14.7	84.1 ± 15.8	0.59
HOS-ADL	91.8 ± 13.3	92.0 ± 13.6	90.9 ± 12.2	0.21
HOS-SS	82.8 ± 23.4	83.3 ± 24.2	80.8 ± 19.6	0.12
iHOT	79.3 ± 22.7	79.0 ± 22.6	75.4 ± 23.6	0.34

The left column represents all eligible patients for the study. The second column represents patients with an intraoperative traction time of <50 min. The third column represents patients with an intraoperative traction time of ≥50 min.

**Table III.** Achievement of the MCID on post-operative PROMs between two cohorts based on intraoperative traction time

	*Short traction (n = 145)*	*Long traction (n = 77)*	*P-value*
6 months post-operatively, delta (%)			
mHSS	20.5 (250.4)	20.7 (252.6)	0.84
HOS-ADL	17.3 (209.0)	15.2 (182.7)	0.61
HOS-SS	22.5 (155.1)	19.1 (132.0)	0.36
iHOT	33.6 (277.6)	32.9 (271.5)	0.96
1 year post-operatively, delta (%)			
mHSS	24.9 (303.5)	20.8 (253.5)	0.07
HOS-ADL	19.3 (233.0)	16.7 (200.8)	0.31
HOS-SS	31.6 (217.8)	27.9 (192.2)	0.25
iHOT	39.2 (323.7)	36.2 (299.5)	0.39
2 years post-operatively, delta (%)			
mHSS	24.8 (303.0)	23.0 (280.9)	0.57
HOS-ADL	19.3 (232.9)	18.4 (222.0)	0.96
HOS-SS	33.1 (228.0)	28.9 (199.5)	0.50
iHOT	40.4 (334.3)	35.1 (290.3)	0.22

The left column represents patients with an intraoperative traction time of <50 min. The right column represents patients with an intraoperative traction time of ≥50 min.

Delta values were based on preoperative PROM scores at each time point.

The achievement of MCID is based on the difference between the reference delta of 8.2 (HSS), 8.3 (HOS-ADL), 14.5 (HOS-SS) and 12.1 (iHOT) [9.]

Four PROMs were analyzed at three post-operative intervals. Thus, each cohort’s average post-operative PROM score and MCID achievement rate were collectively compared three times for each of the four PROM scores. Overall, the cohorts reported comparable scores with no significant difference at any post-operative interval in terms of absolute outcome score. Additionally, there was no significant difference in MCID achievement rate found between the two cohorts at any time point.

With multivariable analysis (MVA), we sought to define the predictors of achieving MCID at 2-year follow-up for patients included in our study. The model was adjusted for factors reaching *P* < 0.20 on univariable comparisons. Table IV reveals the covariates and associated odds ratios (ORs) for the model. Adjusting for potential confounders, the sole predictor for all PROM scores on achieving MCID was traction time [mHHS: OR = 0.88; 95% confidence interval CI (0.80–0.98); *P *= 0.02; HOS-ADL: OR = 0.88; 95% CI (0.79–0.98); *P *= 0.02; HOS-SS: OR = 0.84; 95% CI (0.74–0.96); *P *= 0.01; and iHOT: OR = 0.90; 95% CI (0.82–0.99); *P *= 0.04], while pincer-type FAI resection was found to be highly predictive of MCID achievement for all PROMs except HOS-SS [mHSS: OR = 28.01; 95% CI (1.52–516.08); *P* = 0.02; HOS-ADL: OR = 29.55; 95% CI (1.42–615.39); *P* = 0.03; iHOT: OR = 23.00; 95% CI (1.38–382.06); *P* = 0.03].

**Table IV.** MVA predicting achievement of MCID in PROMs among all patients after hip arthroscopy at 2 years post-operatively

	*Adjusted OR (95% CI)*	*P-value*
mHSS		
Sex	0.71 (0.35–1.41)	0.33
Pincer lesion	0.26 (0.05–1.32)	0.11
Pincer resection	6.48 (1.22–34.62)	<0.05**
Traction time	0.88 (0.83–0.93)	<0.05**
Surgical time	1.01 (0.98–1.03)	0.51
HOS-ADL		
Sex	0.53 (0.27–1.02)	0.06
Pincer lesion	0.09 (0.01–0.64)	<0.05**
Pincer resection	10.77 (1.57–73.94)	<0.05**
Traction time	0.88 (0.84–0.93)	<0.05**
Surgical time	1.01 (0.98–1.04)	0.29
HOS-SS		
Sex	0.50 (0.17–1.50)	0.22
Pincer lesion	0.17 (0.01–2.25)	0.18
Pincer resection	11.29 (0.77–165.47)	0.08
Traction time	0.84 (0.74–0.96)	<0.05**
Surgical time	1.01 (0.99–1.04)	0.33
iHOT		
Sex	0.35 (0.12–1.01)	0.05
Pincer lesion	0.09 (0.01–1.39)	0.08
Pincer resection	23.00 (1.38–382.06)	<0.05**
Traction time	0.90 (0.82–0.99)	<0.05**
Surgical time	1.02 (0.99–1.05)	0.15

ORs are reported such that an OR >1.0 represents an increased odds of improvement in PROMs.

a Multivariate models adjusted for factors with *P*-values of <0.20 on univariate comparisons: sex, pincer lesion, pincer resection, traction time and surgical time.

** A significant difference with *P*-values of <0.05.

## DISCUSSION

While this study’s initial hypothesis anticipated inferior post-operative PROM scores in patients with longer traction durations, the results ultimately suggest no correlation between traction time and PROMs. While cohorts did not significantly differ regarding MCID achievement rates, following MVA, decreased traction duration was found to be a predictor for achieving MCID in all PROM scores. A pincer-type FAI was diagnosed in 27% of patients included in our study, and resection of this deformity was found to highly correlate with MCID achievement. Based on these results, we posit that traction has a time-dependent influence on a patient’s perceived outcome of the arthroscopic intervention; however, traction times in our surgeons and patients were not long enough to have a significant effect on PROMs and MCID.

Traction during hip arthroscopy enables the expansion of the hip joint, thus facilitating the safe passage of instruments into the intra-articular space [1, 2]. However, traction-induced neurologic sequelae are common complications following hip arthroscopy [3, 14–17]. The incidence of these complications remains considerable; Frandsen *et al.* [3] surveyed 100 patients undergoing hip arthroscopy and found 32% reported traction-related problems. The consensus recommendation to avoid traction-based neurologic complications is to keep hip arthroscopy traction duration under 2 h [5, 14, 18–21]. The existing literature on hip arthroscopy traction either focuses on defining the frequency, severity and natural course of these complications or aims to provide technical instruction to prevent iatrogenic injury [1–3, 5, 6, 14, 16, 22–27]. In keeping with this approach, Lo *et al.* [28] examined the relationship between traction duration and neurologic injury after hip arthroscopy. They found that patients with post-operative neurologic symptoms had an average traction duration of 132 min, whereas patients without any neurologic symptoms had an average traction duration of 59 min [28]. Telleria *et al.* [5] found that the maximum traction weight, not the total traction time, is the greatest risk factor for sciatic nerve dysfunction during hip arthroscopy. However, their study was not powered enough to detect traction time differences between the groups. More recently, Bailey *et al.* [1] found that concomitant pudendal and lateral femoral cutaneous nerve palsy may be related to increased traction force and time, even in the setting of low intraoperative traction time (<1 h).

The emphasis on post-operative complications in hip arthroscopy traction literature reflects a historic approach to measuring treatment success as a function of surgical outcomes and complication rates. However, as treatment paradigms shift to a patient-centered focus, patient satisfaction is increasingly considered a key factor for determining treatment success. This has led to increased utilization of PROMs in the orthopedic literature [29–31]. Nonetheless, the literature on hip arthroscopy PROMs as a function of intraoperative traction duration is limited.

This study benefits from several strengths. These strengths include a robust statistical analysis and an overall satisfactory rate of follow-up consistent with other large database studies. Four validated hip-specific PROMs were included, enabling us to demonstrate consistency across a broad range of outcome-reporting tools. Finally, the longitudinal and episodic nature of the follow-up protocol, wherein PROMs were collected and analyzed at 6-month, 1-year and 2-year intervals, provides an insight into the effect of traction duration on both short- and mid-term results of hip arthroscopy.

The present study has several limitations. Foremost, as a single-center study utilizing prospective registry-based data, the generalizability of the results is limited. Second, PROMs were analyzed solely in relation to traction time; traction force was not available for inclusion. Certain studies, such as Telleria *et al.* [5], have identified traction force as a key variable to consider when examining the impact of traction on hip arthroscopy outcomes. Future studies that focus on the impact of traction force on patient-reported outcomes after hip arthroscopy are warranted. A third limitation is that the cohorts differed significantly in terms of rates of pincer-type FAI diagnosis and resection. This likely is a result of forming cohorts based on traction duration. Epidemiologic investigations have shown that pincer-type FAI correlates with longer traction durations [32–34]. We felt that the benefit of having larger cohorts for comparison outweighed the potential downside of these differing baseline characteristics. Moreover, by performing an MVA, we were able to control these factors to limit their impact. Data regarding sequela traction-associated complications were not available for all patients and thus were not included in the analysis. This limits our study as traction-associated post-operative complications could influence patient-reported outcomes. Lastly, while the traction times seen in this study are similar to those reported in other investigations [1, 3, 5, 6, 14, 24], almost all subjects had traction durations well below the generally accepted threshold of 2 h. This limits the conclusions that can be drawn on the relationship between hip arthroscopy PROMs and traction durations of more than 2 h.

## CONCLUSION

There was no difference in PROMs and MCID achievement between longer- and shorter-traction time cohorts. On MVA, a decrease in traction time is predictive of MCID for all PROM scores and pincer-type resection was predictive of MCID for most PROM scores.

## Data Availability

The data supporting the findings of this study are available from the corresponding author upon reasonable request. The data are not publicly available due to privacy or ethical restrictions. Access to the data will be granted upon request.

## References

[1] Bailey TL, Stephens AR, Adeyemi TF et al. Traction time, force and postoperative nerve block significantly influence the development and duration of neuropathy following hip arthroscopy. *Arthroscopy* 2019; 35: 2825–31. 31604499 10.1016/j.arthro.2019.03.062

[2] Flierl MA, Stahel PF, Hak DJ et al. Traction table-related complications in orthopaedic surgery. *J Am Acad Orthop Surg* 2010; 18: 668–75. 21041801 10.5435/00124635-201011000-00004

[3] Frandsen L, Lund B, Grønbech Nielsen T et al. Traction-related problems after hip arthroscopy. *J Hip Preserv Surg* 2017; 4: 54–9. 28630721 10.1093/jhps/hnw044 PMC5467411

[4] Larson CM, Clohisy JC, Beaulé PE et al. Intraoperative and early postoperative complications after hip arthroscopic surgery: a prospective multiscenter trial utilizing a validated grading scheme. *Am J Sports Med* 2016; 44: 2292–8. 27311412 10.1177/0363546516650885

[5] Telleria JJM, Safran MR, Gardi JN et al. Risk of sciatic nerve traction injury during hip arthroscopy—is it the amount or duration? An intraoperative nerve monitoring study. *J Bone Joint Surg Am* 2012; 94: 2025–32. 23052834 10.2106/JBJS.K.01597

[6] Martin HD, Palmer IJ, Champlin K et al. Physiological changes as a result of hip arthroscopy performed with traction. *Arthroscopy* 2012; 28: 1365–72. 22920287 10.1016/j.arthro.2012.04.139

[7] Tonotsuka H, Sugiyama H, Tanaka D et al. Postoperative creatine kinase elevation following hip arthroscopy and associated risk factors. *Acta Orthop Traumatol Turc* 2019; 53: 397–401. 31537432 10.1016/j.aott.2019.08.011 PMC6938993

[8] Martin RRL, Philippon MJ. Evidence of validity for the hip outcome score in hip arthroscopy. *Arthroscopy* 2007; 23: 822–6. 17681202 10.1016/j.arthro.2007.02.004

[9] Nwachukwu BU, Fields K, Chang B et al. Preoperative outcome scores are predictive of achieving the minimal clinically important difference after arthroscopic treatment of femoroacetabular impingement. *Am J Sports Med* 2017; 45: 612–9. 27765733 10.1177/0363546516669325

[10] Martin RRL, Kelly BT, Philippon MJ. Evidence of validity for the hip outcome score. *Arthroscopy* 2006; 22: 1304–11. 17157729 10.1016/j.arthro.2006.07.027

[11] Byrd JWT . Hip arthroscopy: patient assessment and indications. *Instr Course Lect* 2003; 52: 711–9. 12690896

[12] Nwachukwu BU, Chang B, Beck EC et al. How should we define clinically significant outcome improvement on the iHOT-12? *HSS J* 2019; 15: 103–8. 31327939 10.1007/s11420-018-9646-0 PMC6609659

[13] Ueland TE, Carreira DS, Martin RRL. Substantial loss to follow-up and missing data in national arthroscopy registries: a systematic review. *Arthroscopy* 2021; 37: 761–70.e3. 32835814 10.1016/j.arthro.2020.08.007

[14] Kern MJ, Murray RS, Sherman TI et al. Incidence of nerve injury after hip arthroscopy. *J Am Acad Orthop Surg* 2018; 26: 773–8. 30180092 10.5435/JAAOS-D-17-00230

[15] Habib A, Haldane CE, Ekhtiari S et al. Pudendal nerve injury is a relatively common but transient complication of hip arthroscopy. *Knee Surg Sports Traumatol Arthrosc* 2018; 26: 969–75. 29119283 10.1007/s00167-017-4783-4

[16] Byrd JWT . Hip arthroscopy. *J Am Acad Orthop Surg* 2006; 14: 433–44. 16822891 10.5435/00124635-200607000-00006

[17] Weber AE, Harris JD, Nho SJ. Complications in hip arthroscopy: a systematic review and strategies for prevention. *Sports Med Arthrosc* 2015; 23: 187–93. 26524553 10.1097/JSA.0000000000000084

[18] Sampson TG . Complications of hip arthroscopy. *Clin Sports Med* 2001; 20: 831–6. 11675890 10.1016/s0278-5919(05)70288-x

[19] Ilizaliturri VM . Complications of arthroscopic femoroacetabular impingement treatment: a review. *Clin Orthop Relat Res* 2009; 467: 760–8. 19018604 10.1007/s11999-008-0618-4 PMC2635434

[20] Mason JB, McCarthy JC, O’Donnell J et al. Hip arthroscopy: surgical approach, positioning, and distraction. *Clin Orthop Relat Res* 2003; 406: 29–37. 10.1097/01.blo.0000043041.84315.cc 12578997

[21] Clarke MT, Arora A, Villar RN. Hip arthroscopy: complications in 1054 cases. *Clin Orthop Relat Res* 2003; 406: 84–8. 10.1097/01.blo.0000043048.84315.af 12579004

[22] Kocaoʇlu H, Başarir K, Akmeşe R et al. The effect of traction force and hip abduction angle on pudendal nerve compression in hip arthroscopy: a cadaveric model. *Arthroscopy* 2015; 31: 1974–80.e6. 26033463 10.1016/j.arthro.2015.03.040

[23] Elsaidi GA, Ruch DS, Schaefer WD et al. Complications associated with traction on the hip during arthroscopy. *J Bone Joint Surg Br* 2004; 86: 793–6. 15330016 10.1302/0301-620x.86b6.14426

[24] Lall AC, Saadat AA, Battaglia MR et al. Perineal pressure during hip arthroscopy is reduced by use of Trendelenburg: a prospective study with randomized order of positioning. *Clin Orthop Relat Res* 2019; 477: 1851–7. 31261260 10.1097/CORR.0000000000000804 PMC7000000

[25] Papavasiliou AV, Bardakos NV. Complications of arthroscopic surgery of the hip. *Bone Joint Res* 2012; 1: 131–44. 23610683 10.1302/2046-3758.17.2000108 PMC3629445

[26] Nakano N, Khanduja V. Complications in hip arthroscopy. *Muscles Ligaments Tendons J* 2016; 6: 402–9. 28066747 10.11138/mltj/2016.6.3.402 PMC5193532

[27] Pailhé R, Chiron P, Reina N et al. Pudendal nerve neuralgia after hip arthroscopy: retrospective study and literature review. *Orthop Traumatol Surg Res* 2013; 99: 785–90. 24080353 10.1016/j.otsr.2013.07.015

[28] Lo Y-P, Chan Y-S, Lien L-C et al. Complications of hip arthroscopy: analysis of seventy three cases. *Chang Gung Med J* 2006; 29: 86–92. 16642731

[29] Hamilton DF, Lane JV, Gaston P et al. What determines patient satisfaction with surgery? A prospective cohort study of 4709 patients following total joint replacement. *BMJ Open* 2013; 3: e002525. 10.1136/bmjopen-2012-002525 PMC3641464 23575998

[30] Kim S, Duncan PW, Groban L et al. Patient-reported outcome measures (PROM) as a preoperative assessment tool. *J Anesth Perioper Med* 2017; 4: 274–81. 29333531 PMC5766034

[31] Fidai MS, Saltzman BM, Meta F et al. Patient-reported outcomes measurement information system and legacy patient-reported outcome measures in the field of orthopaedics: a systematic review. *Arthroscopy* 2018; 34: 605–14. 29096979 10.1016/j.arthro.2017.07.030

[32] Kapron AL, Karns MR, Aoki SK et al. Patient-specific parameters associated with traction in primary and revision hip arthroscopic surgery. *Orthop J Sports Med* 2018; 6: 2325967118807707. 10.1177/2325967118807707 PMC6243416 30480019

[33] Ellenrieder M, Tischer T, Bader R et al. Patient-specific factors influencing the traction forces in hip arthroscopy. *Arch Orthop Trauma Surg* 2017; 137: 81–7. 27695971 10.1007/s00402-016-2572-z

[34] Matsuda DK, Gupta N, Hanami D. Hip arthroscopy for challenging deformities: global pincer femoroacetabular impingement. *Arthrosc Tech* 2014; 3: e197–204. 24904760 10.1016/j.eats.2013.09.021 PMC4044509

